# Preferences for the sequencing of first-line systemic treatments in metastatic hormone receptor-positive, HER2-negative breast cancer

**DOI:** 10.3389/fonc.2023.1181375

**Published:** 2023-10-18

**Authors:** Layal Al Mahmasani, Ghid Amhaz, Reine Abou Zeidane, Nathalie Chamseddine, Taha Hatab, Saad Sabbagh, Maya Charafeddine, Hazem I. Assi

**Affiliations:** ^1^ Naef K. Basile Cancer Institute, Division of Hematology and Oncology, Department of Internal Medicine, American University of Beirut Medical Center, Beirut, Lebanon; ^2^ Department of Obstetrics and Gynecology, American University of Beirut Medical Center, Beirut, Lebanon; ^3^ Department of Internal Medicine, American University of Beirut Medical Center, Beirut, Lebanon

**Keywords:** metastatic breast cancer, hormone therapy, chemotherapy, targeted therapies, sequence, preferences

## Abstract

**Introduction:**

Metastatic breast cancer (MBC) is a diverse disease. Therapeutic options include hormonal therapy, chemotherapy, and targeted therapies. The optimal treatment sequence for patients with hormone receptor-positive (HR-positive), HER2-negative metastatic breast cancer remains unknown.

**Methods:**

This was a retrospective and prospective study. The data was collected from the medical records of patients in a tertiary healthcare center in Lebanon between the years 2016 and 2019, and patients were followed up for a 3-year duration. The main aim was to identify oncologists’ preferences in the choice and sequence of treatment for newly diagnosed and/or recurrent cases of HR-positive, HER2-negative MBC.

**Results:**

A total of 51 patients were included. 24 patients received chemotherapy, while 27 received endocrine therapy as first-line treatment after a diagnosis of MBC, with a median overall survival (OS) of 13 months and a median progression-free survival (PFS) of 12 months after first-line treatment with chemotherapy, compared to 27 months and 18 months with endocrine therapy. A higher percentage of patients have received chemotherapy in the first-line setting compared to the data reported in the literature, with the choice being multifactorial.

**Conclusion:**

Factors to consider in MBC management include the choice of first-line treatment, the optimal sequence of treatment, and the combination of available treatment options.

## 1 Introduction

Breast cancer is the most common cancer in women and a leading cause of cancer-related deaths worldwide. The annual incidence of newly diagnosed breast cancer is approximately 124.9 per 100,000 women (1). In 2023, 297,790 new cases and 43,170 deaths occurred among US women (2). In Lebanon, it accounts for 38.2% of female tumors, higher than in regional and Western countries (3).

Breast cancer is a heterogeneous, phenotypically diverse disease that includes several biological subtypes characterized by different responses to therapy. Classification of this disease into clinical phenotypes depends on the presence or absence of hormone receptors [estrogen receptor (ER) and progesterone receptor (PR)] and/or human epithelial receptor 2 (HER2/neu). Hormone receptor (HR)-positive breast cancers account for 75 percent of all breast cancer cases. This disease is classified by stage, including local, advanced, regionally advanced, and metastatic. Metastatic breast cancer varies depending on the metastasis site, organ involvement, and prognostic characteristics (4). It occurs in recurrent or progressive cases (85-90%) or *de novo* cases (5-10%) (5).

Early breast cancer treatment is generally curative, while metastatic treatment focuses on palliative control of symptoms, prolonging survival, and minimizing adverse effects. Treatment selection is complex and depends on patient characteristics, disease-related features, pathological characteristics, previous treatment response and disease-free survival (6), in addition to personal and oncologists’ preferences based on training, expertise, availability, and accessibility to certain agents. Treatment options available for breast cancer include hormonal and/or chemotherapeutic agents such as taxanes, anthracyclines, vinorelbine, and capecitabine, in addition to pathway-targeting drugs such as CDK4/6 inhibitors and mTOR antagonists. Multiple guidelines are available for the treatment of breast cancer; however, there is no consensus on the best sequence of agents to be utilized for the treatment of metastatic HR-positive breast cancer (7).

HR-positive, HER2/neu-negative MBC cases make up 67-70% of all MBC cases (8), with endocrine therapy being the initial treatment. This includes selective estrogen receptor modulators like tamoxifen, antagonists like fulvestrant, and aromatase inhibitors like letrozole and anastrozole. Treatment depends on age and menopausal status (9). Studies have shown improvements in survival with CDK4/6 inhibitors, classified as first-line and second-line treatments. Palbociclib, a CDK4/6 inhibitor, showed longer progression-free survival in PALOMA 2 and PALOMA 3 trials (10). The MONALEESA-2 trial found that ribociclib and letrozole combined with AI improved progression-free survival and overall survival in hormone-positive, HER2-negative MBC patients (11). MONARCH 3 found that abemaciclib, a CDK4/6 inhibitor, increased response rates and reduced disease progression risk in women with MBC. However, data on OS and PFS is still immature (12).

Given the above-mentioned data regarding different available therapeutic options, endocrine therapy remains the first option for metastatic hormone receptor-positive, HER2-negative MBC patients without visceral crisis or the need for a rapid response in severely symptomatic patients (13). However, selecting the best agent remains challenging due to the lack of trials comparing combinations; as such, treatment guidelines for MBC remain unclear. Therefore, the purpose of the present analysis was to identify preferences in the choice of treatment (first- and second-line) by oncologists for newly diagnosed and/or recurrent MBC and to analyze differences or similarities in outcomes accordingly. In addition, we aimed to assess whether different choices of treatment and sequences influenced significant health-related outcomes, including progression-free survival and adverse outcomes.

## 2 Methods

This was a retrospective chart review and prospective study with a 3-year follow-up period that used medical records to identify patients with MBC and then further classified them based on disease characteristics and receptor status, treatment use and associated response, morbidity, and mortality data.

The medical charts of patients who were treated at the American University of Beirut Medical Center for MBC, whether newly diagnosed or recurring with metastatic disease, between the years 2016 and 2019, were reviewed.

The included patients were followed prospectively for 3 years by reviewing their clinical charts to identify response to treatment, documented adverse events, and the need to switch or discontinue therapy, with a calculation of progression-free survival. Note that ER and PR are considered positive when positive tumor cell nuclei of any intensity are present in >1% of tumor cells.

The inclusion criteria were patients older than 18 years of age with hormone receptor-positive, HER2-negative MBC who presented with a new diagnosis of MBC (recurrence or *de novo*). The exclusion criteria were patients with non-MBC, patients with HER2-positive MBC, or patients diagnosed with MBC prior to January 2016. Oral consent was obtained from the patients who were alive.

Data were collected, entered, and analyzed using the SPSS software. Demographics, presentation, staging, treatment sequence, and treatment results throughout the three years of follow-up were analyzed. Progression-free survival was calculated and plotted using Kaplan-Meier survival analysis.

### 2.1 Results

A total of 836 charts of patients diagnosed with breast cancer between January 2016 and December 2019 were screened. 93 patients were eligible for inclusion in the study. We were able to follow prospectively and have all the needed data since the diagnosis of metastatic disease up to 3 years of follow-up for a total of 51 patients out of the 93.

The baseline characteristics of the included patients are presented in **Table 1**. Overall, 45% of the patients were previously healthy, 68% were postmenopausal, and 68% were never smokers. 78% of the patients had invasive ductal carcinoma on pathology, while 18% had invasive lobular carcinoma. 53% of patients had *de novo* MBC, whereas 47% of patients had metastatic disease at relapse. The proportion of metastatic disease is relatively high, and this is correlated with the deteriorating financial and economic status and lack of health insurance in Lebanon, which is reflected in delayed screening. Among those who had metastatic disease at relapse, 60%, 53%, 47%, and 51% of the patients had undergone surgery, received radiation therapy, endocrine therapy, and/or chemotherapy (whether adjuvant or neoadjuvant), respectively. As such, among all 51 patients, 53% were endocrine therapy naïve, and 55% were chemotherapy naïve.

**Table 1:** Baseline characteristics for all patients.

Variables		Frequency (total 51)	Percentage (%)
**Age, median (range)**		56.5 (28-88)	
**Past medical History**	Healthy Hypertension Diabetes mellitus Dyslipidemia Others	23 7 2 1 18	45 14 4 2 35
**Smoking status**	Never smoker Current smoker Ex-smoker Unknown	35 10 5 1	68 20 10 2
**Menopausal status**	Premenopausal Perimenopausal Postmenopausal	12 4 35	23 9 68
**Family History of breast cancer**	Yes No	9 42	18 82
**Pathology**	IDC ILC Other	40 9 2	78 18 4
**ER Receptor status**	Strongly positive Weakly positive	49 2	96 4
**PR Receptor status**	Positive Negative	49 2	96 4
***De novo* metastatic disease**		27	53
**Metastatic disease at relapse**		24	47
**Treatments prior to diagnosis of metastatic disease**	Surgery Radiation therapy Endocrine therapy Chemotherapy (adjuvant or neoadjuvant)	30 27 24 26	60 53 47 51
**ET naïve**		27	53
**CT naïve**		28	55
**Death rate**	Alive Dead Missing	34 15 2	66 30 4

24 patients received chemotherapy as first-line treatment, whereas 27 patients received endocrine therapy as first-line treatment after a diagnosis of metastatic disease. The baseline characteristics of patients in each group are shown in **Table 2**. The reasons for selecting chemotherapy as the first-line treatment are shown in **Table 3**. and are mainly related to significant disease burden as reported by the treating physician, either due to significant visceral disease, whether in the liver or lungs, or due to symptomatic disease mainly in the lungs or bone, causing severe respiratory symptoms or severe bony pain. Among the 24 patients who received chemotherapy as first-line treatment, six were then shifted to endocrine therapy as sequential treatment and maintenance therapy prior to disease progression. Second- and third-line treatments are described in **Table 4**.

**Table 2:** Baseline Characteristics stratified by type of first-line treatment received.

		Chemotherapy group (n=24)	Endocrine therapy group (n=27)
**Age, median (range)**		56 (31-82)	62 (39-85)
**Menopausal status**	Premenopausal Perimenopausal Postmenopausal	7 1 16	5 3 19
***De novo* metastatic disease**		14	10
**Metastatic disease at relapse**		10	17
**Treatments prior to diagnosis of metastatic disease**	Endocrine therapy Chemotherapy	11 10	23 16

**Table 3:** Reasons for the selection of chemotherapy as first-line treatment among the 24 patients.

Reason		Frequency	Percentage
**High, symptomatic disease burden in**	Liver Lungs Bone Multiple sites	5 5 2 3	21 21 8 12
**Unclear reason**		9	38

**Table 4:** Second line and third line treatments.

2nd line treatments	
**Endocrine and Targeted therapy**	**7**
**Endocrine therapy**	**5**
**Chemotherapy**	**19**
Capecitabine	**5**
Doxorubicin	**1**
Carboplatin/Gemzar	**1**
Navelbine	**1**
Navelbine/Capecitabine	**5**
Eribulin	**1**
Cyclophosphamide/doxorubicin/Taxotere	**3**
3rd line treatments	
**Endocrine and Targeted therapy**	**2**
**Endocrine therapy**	**5**
**Chemotherapy**	**14**
Capecitabine	**1**
Doxorubicin	**1**
Carboplatin/Gemzar	**1**
Navelbine	**1**
Navelbine/Capecitabine	**1**
Eribulin	**3**
Cyclophosphamide/Doxorubicin/Taxotere	**2**
Taxotere	**2**
Taxotere/Cyclophosphamide	**1**
Taxol	**1**

**Figure 1:**
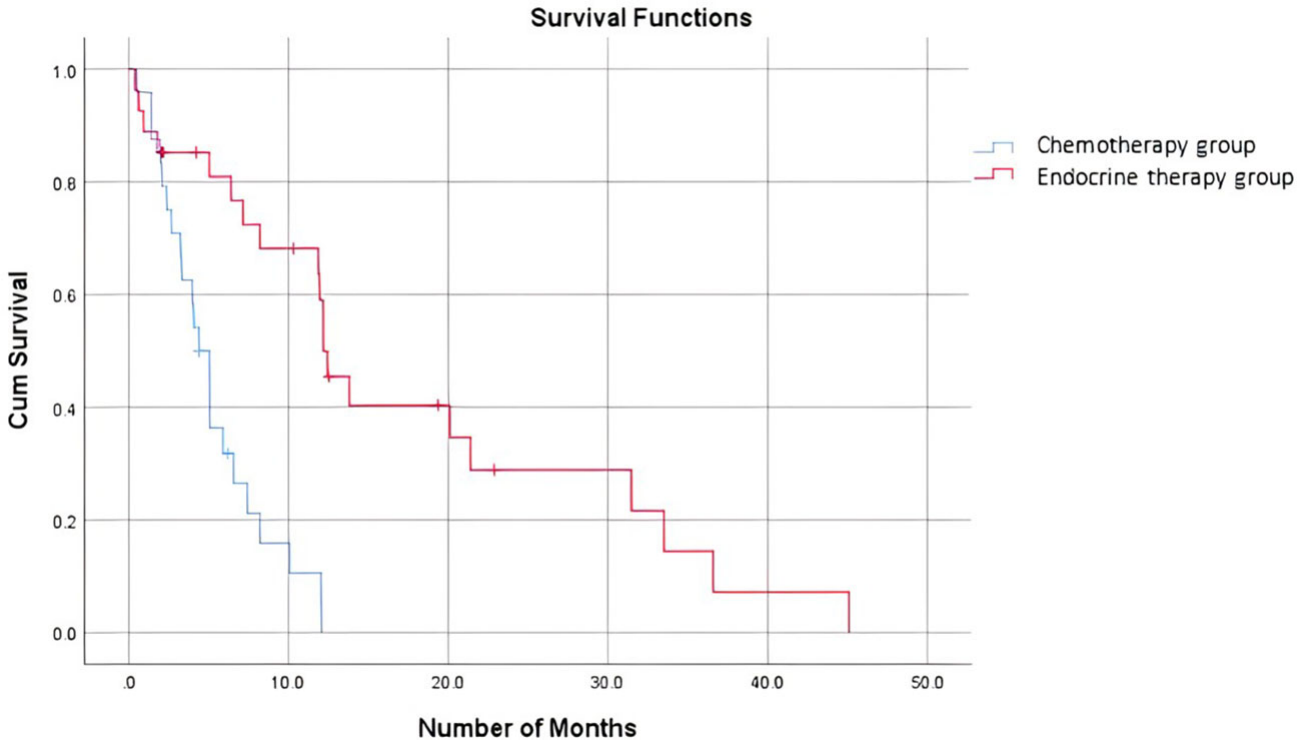
Time on first line treatment.

**Figure 2:**
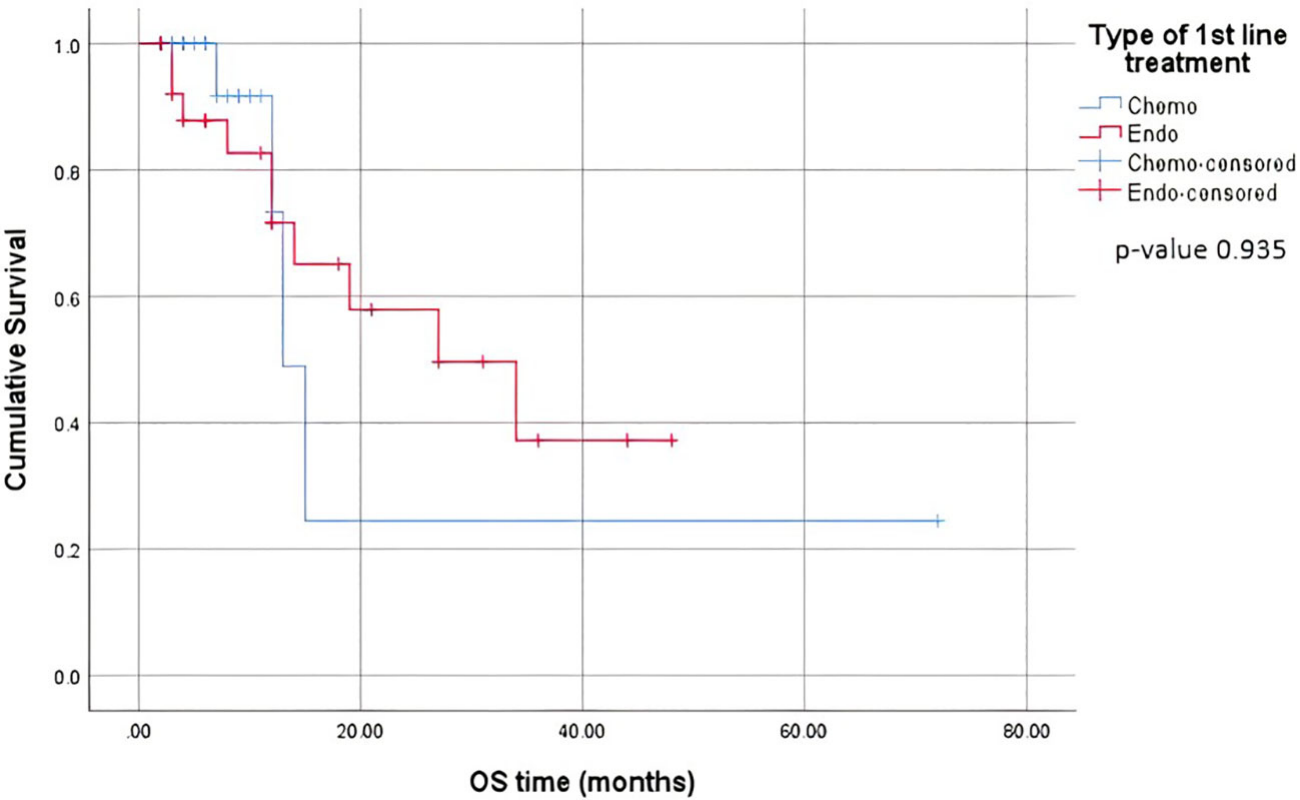
Overall Survival on first line treatment.

**Figure 3:**
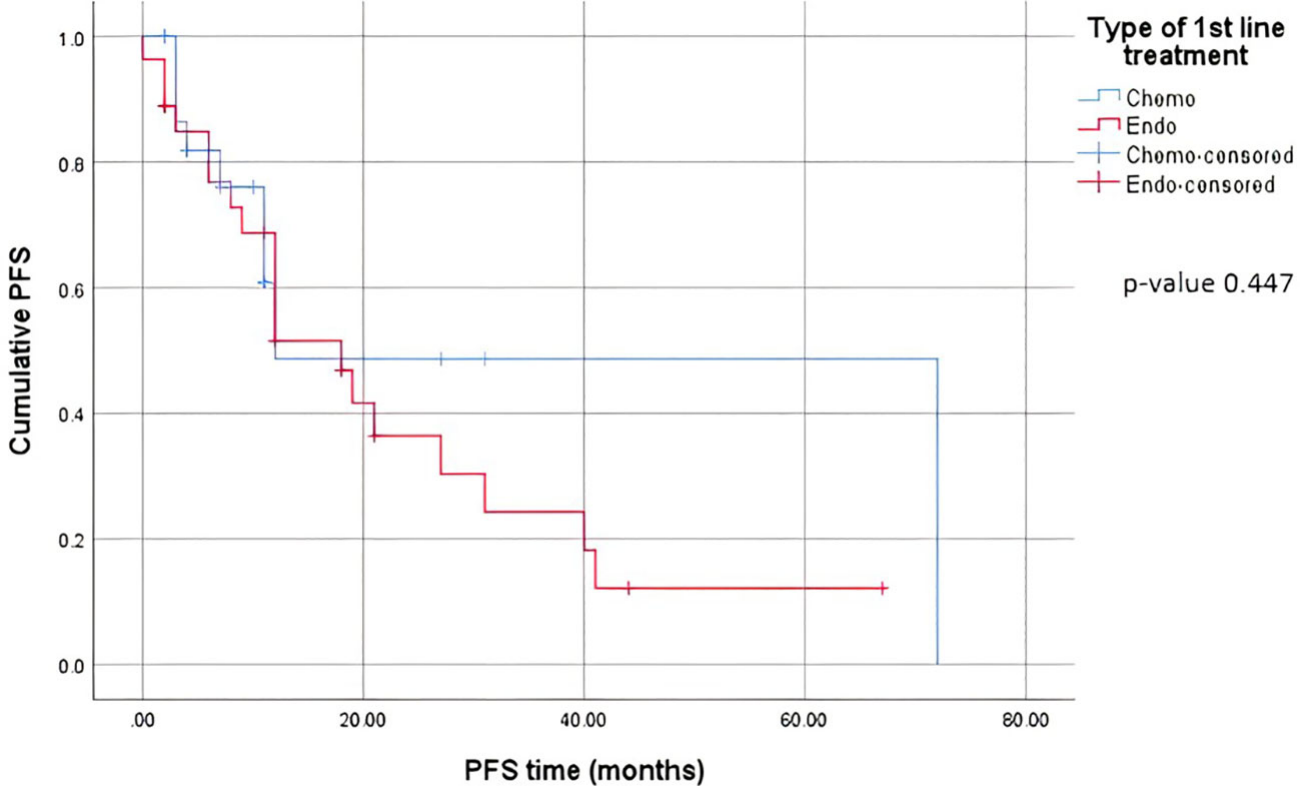
Progression-Free survival on first line treatment.

When comparing the duration of first-line treatment (i.e., time since initiation of first-line treatment until time of progression), we found that patients who were on chemotherapy had a mean duration of treatment equivalent to 5.25 months, compared to 17.69 months for those on endocrine therapy (**Figure 1**). Regarding adverse events (shown in **Table 5**.), the available data are limited. We noted similar reported adverse events in both groups; however, most adverse events were unspecified and not graded in terms of severity. Eleven patients discontinued treatment due to disease progression. The median overall survival after first-line treatment with chemotherapy was 13 months, compared to 27 months with endocrine therapy, with a p-value of 0.935 (**Figure 2**). Regarding progression-free survival (PFS), the median PFS for the chemotherapy group was 12 months compared to 18 months in the endocrine therapy group, with a non-significant p-value of 0.447 (**Figure 3**).

**Table 5:** Toxicities reported among patients in the first-line setting.

Adverse events	Chemotherapy group (n=24)	Endocrine therapy group (n=27)
**Anemia**	2 (8.3%)	2 (7.4%)
**Thrombocytopenia**	0	1 (3.7%)
**Unspecified**	16 (66.6%)	18 (66.7%)

Looking further into patients who were started on endocrine therapy as the first line therapy, we found that 17 out of 27 patients were started on both endocrine and targeted therapies [ribociclib (2), palbociclib (14)] and 10 on endocrine therapy alone. In this study, there was no significant difference in the progression-free survival rates between the 2 groups (16.3 months for the endocrine alone group and 18.4 months for the combination group).

### 2.2 Discussion

MBC treatment is heterogeneous. This heterogeneity is multifactorial because it depends on the following: (a) physician preference and expertise, (b) patient preference, (c) financial limitations, (d) disease status, and (e) a combination of previous factors. The effect of this variability on patient outcomes has not been well understood or studied. In this study, we examined treatment sequencing among patients who were diagnosed with MBC (*de novo* and metastatic at relapse) and its effect on patient outcomes.

Regarding the choice of first-line treatment, 24 of 51 patients (47%) received chemotherapy, compared to 27 out of 51 patients (53%) who received first-line endocrine therapy. In comparison to other studies, a higher proportion of patients received chemotherapy in the first-line setting at our institution. Basile et al. found that, among hormone receptor-positive, HER2-negative MBC patients, 31% received first-line chemotherapy, 49% received endocrine therapy alone, and 20% received endocrine therapy plus a CDK4/6 inhibitor (15). Data from previous years and older studies, however, revealed a trend similar to that found in our study, where a retrospective German study of patients treated between 2002 and 2004 showed that less than half of women with hormone-receptor-positive MBC (48%) received ET in any line of treatment (14). Another study reported by Swallow et al. in 2014 described the fact that despite combination chemotherapy being the preferred first-line treatment for MBC, irrespective of the number of organs involved, hardly any patient received endocrine therapy only (16). It has also been reported that, in general, the percentage of hormone receptor-positive patients in chemotherapy studies ranges from 70 to 80%, whereas visceral involvement is present in approximately 50-80% of patients undergoing chemotherapy and in approximately 50% of those treated with endocrine therapy (17).

Possible reasons for this variation in the choice of first-line treatment and initiation of first-line chemotherapy may be the following:

1- Confounding visceral metastasis and visceral crisis: Oncologists might be confused regarding these two terms and indications for treatment because visceral crisis rather than visceral disease is considered an indication for chemotherapy. Confounding these two conditions may result in not even considering endocrine therapy in any case of visceral involvement (17).
2- Patient-related factors through the eyes of the treating physician: Oncologists may tend to initiate chemotherapy as a first-line treatment for young fit patients. In a Dutch study of metastatic hormone receptor-positive breast cancer patients treated in eight mostly non-academic institutions, it has been shown that 24% received initial chemotherapy, and these patients tended to be younger, have fewer comorbidities, were more often exposed to adjuvant chemotherapy and endocrine therapy, and were more likely to have visceral metastases (18).
3- Patient preference: Treatment-related adverse events and toxicities play a major role in the selection of treatment, especially in metastatic cancers, where the aim of treatment is palliation and prolonging disease progression rather than tumor shrinkage. In a cross-sectional survey of 360 post-menopausal women from the US and Europe, endocrine therapy has been shown to be associated with better health-related quality of life, greater satisfaction with treatment, better feelings about side effects, less bothersome treatment side effects, and less activity impairment than patients who receive chemotherapy (19). Patients need to be aware of the pros and cons of each type of therapy and accordingly decide with the treating oncologist which type of therapy to pursue first in a well-informed manner. This is highly dependent on the patient’s level of education and understanding of the disease.
4- In Lebanon, oncologists are extremely limited in their treatment options because expensive and essential oncology drugs are lacking. Standards of care are frequently not observed, putting cancer patients in critical and life-threatening situations (20). Based on our experience at our medical center and the failure of solution implementation by the government, public medical financial support is severely lacking, relying primarily on private donations and limiting access to healthcare.

Receptor conversion is a well-documented event in progressive MBC, which results in HR and HER2 status discordance between the primary tumor and metastatic site (21). An increasing number of guidelines recommend a re-biopsy of metastatic breast cancer to determine the receptor status and molecular subtype (22). In a study by Shen et al., nearly half of the patients were on regimens containing endocrine therapy as the first-line treatment according to the receptor status of metastatic sites when ER or PR changed from negative to positive, with post-recurrence survival significantly better than that based on the primary receptor status after chemotherapy alone. However, there was no statistically significant better prognosis in patients in the HER2 primary negative metastatic positive subgroup who received HER2-targeted therapy as multiline treatment (22). In another study by Lu et al., the 2-year post-recurrence survival rates of treatment-changed and treatment-unchanged patients were 48.1% and 90.0% respectively (23).

When comparing the duration of first-line treatment, we found that patients who were on chemotherapy had a mean duration of treatment equivalent to 5.25 months, compared to 17.69 months for those on endocrine therapy. This shows that first-line chemotherapy alone was associated with worse progression-free survival (PFS). This is consistent with data proven in several studies where it was shown that patients who received endocrine therapy as a first-line treatment combined with CDK4/6 inhibitors had a better PFS than those who received the same treatment in the second-line setting (15). A study by Karacin et al. showed that subsequent ET could be as effective as CT in patients whose disease progressed under ET + CDKi treatment, and better PFS could be obtained with the subsequent everolimus-based therapy than with monotherapy ET after first-line CDKi (2, 24). Nonetheless, we should keep in mind a potential bias and interpret the results carefully, knowing that in general, patients who are started on chemotherapy as the first-line treatment tend to have more advanced disease and an impending visceral crisis, thereby inherently having a worse prognosis. Endocrine monotherapy is considered an effective treatment option, especially for patients whose disease course is more indolent, i.e., a disease-free interval prolonged beyond two years, or for patients presenting with *de novo* low-burden and non-visceral metastatic disease (25). In addition, resistance to endocrine therapy is key to the management of MBC. Despite the benefits of endocrine therapy, drug resistance frequently develops, and the median progression-free survival or time to progression ranges from 6-11 months for first-line endocrine therapies (26). A multicenter, phase 3 trial comparing the use of a combination of CDK4/6i abemaciclib with ET to chemotherapy capecitabine in HR-positive patients showed no superiority of ET to chemotherapy, yet better tolerated side effects and improved quality of life with ET (27).

The present study has some limitations, such as its retrospective and prospective design, its single-center design, and the small sample size. More specifically, due to the paucity of patients in each study group (endocrine therapy vs. chemotherapy as the first-line treatment), the results need to be interpreted with caution. Moreover, the choice of treatment is influenced by several factors that cannot be targeted individually, such as clinician preference in addition to patient and disease characteristics. Nevertheless, treatment-sequence visualization remains a key concept that can enhance the capacity to effectively conceptualize treatment patterns and patient outcomes (28).

#### 2.2.1 Current situation in Lebanon

Contrary to what is happening in industrialized countries, and to help overcome drug shortages and for the sake of their patients, oncologists are tempted by unorthodox approaches such as using on/off prescriptions, switching between different brands of the same drug class, using suboptimal drug dosages, and even deviating from the recommended mode of intake of some drugs (20).

Suggested solutions include the advancement of laboratory diagnostic research and the careful selection of patients. Increased funding will be required to cover potential expenses and to use innovative and recommended therapies again in Lebanon.

### 2.3 Conclusion

The essential concerns to take into consideration in the management of hormone receptor-positive breast cancer include the choice of first-line treatment, the optimal sequence of treatment, and the combination of available treatment options. In patients with metastatic disease with a low tumor burden, it is advisable to focus on delaying disease progression with acceptable treatment-related toxicity. Practicing personalized or precision medicine remains key in the management of such heterogeneous diseases given the multiple factors that can influence the choice of treatment.

### 2.4 Summary points

• Breast cancer is the most common type of cancer in women and one of the leading causes of cancer-related death in women worldwide.
• The optimal sequence of treatment for patients with hormone receptor-positive, HER2-negative MBC remains unknown.
• The essential concerns to consider in the management of hormone receptor-positive breast cancer include the choice of first-line treatment, the optimal sequence of treatment, and the combination of available treatment options.

## Data availability statement

The raw data supporting the conclusions of this article will be made available by the authors, without undue reservation.

## Ethics statement

The studies involving humans were approved by Institutional Review Board at the American University of Beirut Medical Center. The studies were conducted in accordance with the local legislation and institutional requirements. The ethics committee/institutional review board waived the requirement of written informed consent for participation from the participants or the participants’ legal guardians/next of kin because Oral informed consent was obtained from included patients.

## Author contributions

HA and LM contributed to conception and design of the study. GA and RAZ organized the database. MC and NC performed the statistical analysis. TH, SS, and NC wrote the first draft of the manuscript. LM, GA, RAZ, TH, and SS wrote sections of the manuscript. All authors contributed to the article and approved the submitted version.
